# A small molecule inhibitor for ATPase activity of Hsp70 and Hsc70 enhances the immune response to protein antigens

**DOI:** 10.1038/srep17642

**Published:** 2015-12-03

**Authors:** Kyung-Hwa Baek, Haiying Zhang, Bo Ryeong Lee, Young-Guen Kwon, Sang-Jun Ha, Injae Shin

**Affiliations:** 1 National Creative Research Initiative Center for Biofunctional Molecules, Department of Chemistry, Yonsei University, Seoul 03722, Korea; 2 Department of Biochemistry, College of Life Science & Biotechnology, Yonsei University, Seoul 03722, Korea

## Abstract

The ATPase activities of Hsp70 and Hsc70 are known to be responsible for regulation of various biological processes. However, little is known about the roles of Hsp70 and Hsc70 in modulation of immune responses to antigens. In the present study, we investigated the effect of apoptozole (Az), a small molecule inhibitor of Hsp70 and Hsc70, on immune responses to protein antigens. The results show that mice administered with both protein antigen and Az produce more antibodies than those treated with antigen alone, showing that Az enhances immune responses to administered antigens. Treatment of mice with Az elicits production of antibodies with a high IgG2c/IgG1 ratio and stimulates the release of Th1 and Th2-type cytokines, suggesting that Az activates the Th1 and Th2 immune responses. The observations made in the present study suggest that inhibition of Hsp70 and Hsc70 activities could be a novel strategy designing small molecule-based adjuvants in protein vaccines.

Adjuvants stimulate host immunity to administered vaccine antigens and, as a result, are clinically useful for prevention of infection by pathogenic bacteria and virus^1^,^2^,^3^,^4^. Specifically, adjuvants enable the use of smaller numbers and doses of vaccine injections by enhancing immune responses to vaccines. Despite recent progress made in their discovery^5^,^6^,^7^, only a small number of small molecule-based adjuvants have been approved for clinical use. Thus, a greater effort needs to be made for the development of efficacious small molecule adjuvants^8^,^9^.

The Hsp70 protein family is known to play diverse roles in biological processes^10^,^11^. The two major members of this family, constitutive Hsc70 and inducible Hsp70, are composed of an N-terminal ATPase domain (or a nucleotide binding domain), which binds and catalyzes the hydrolysis of ATP to ADP, and a C-terminal substrate binding domain, which associates with peptide/protein substrates. The two domains are functionally coupled in such a way that hydrolysis of ATP by ATPase activity induces conformational changes in the adjacent substrate binding domain of the proteins. Alterations of the substrate binding domain lead to increases in binding affinities of substrates^12^.

A representative function of the Hsp70 family is chaperone activity such as protein folding, suppression of aggregation of denatured proteins, removal of misfolded proteins and regulation of assembly/disassembly of protein complexes^13^,^14^,^15^,^16^. In addition, members of this protein family are also known to be involved in suppression of apoptotic cell death through multiple anti-apoptotic processes^17^,^18^,^19^,^20^,^21^. In particular, their suppression of cancer cell death leads to tumor cell survival and progression. Because of their pathological significance, small molecule-based inhibitors of these proteins have been exploited for use as potential therapeutic agents and/or chemical probes^22^,^23^,^24^. For example, apoptozole (Az, Fig. 1), which inhibits Hsp70 and Hsc70 activities by binding to ATPase domains^15^,^20^,^21^, and phenylethynesulfonamide (PES), which binds to the C-terminus of Hsp70 but not to Hsc70^25^, display anticancer activities. In addition, inhibitors of these proteins cause a reduction in the accumulation of misfolded tau and promote membrane trafficking of mutant cystic fibrosis transmembrane conductance regulator (CFTR) in cystic fibrosis cells^15^,^26^.

Although extensive investigations of the chaperone and anti-apoptotic activities of members of the Hsp70 family have been performed, only a few studies focusing on Hsp70 associated immune responses have been reported^27^,^28^,^29^. For instance, Hsp70 was found to block lipopolysaccharide (LPS)-induced generation of inflammatory cytokines by suppressing NF-κB activation^27^. In addition, a reduced level of Hsp70 expression in cancer cells triggers specific immune responses, presumably by enhancing cell death *in vivo*^28^. On the other hand, secreted Hsp70 is known to function as both chaperone and adjuvant by promoting the uptake of antigenic peptides into antigen presenting cells and by activating adaptive immune response of neighbor cells^29^. It is likely that the secreted and intracellular Hsp70 may have the different effects on the immune response. The observations made in the previous studies suggest that small molecule inhibitors of Hsp70 and Hsc70 have the potential of modulating the immune system. However, the effect of Hsp70 and Hsc70 inhibitors on immune responses has not been explored to date.

In the study described below, we carried out the first investigation of the effect of a small molecule inhibitor of Hsp70 and Hsc70 on the immune response to protein antigens. The results of the effort show that more antibodies specific to antigens are generated in mice administered with both antigen and Az than those treated with antigen alone. This finding demonstrates that Az enhances the immune response to administered antigens and, thus, it acts as a positive regulator in production of antigen-specific antibodies. Importantly, the results suggest that targeting Hsp70 and Hsc70 could serve as a novel strategy to design small molecule-based adjuvants, which enhance host immunity to vaccinated antigens.

## Results and Discussion

### Az enhances antibody production in mice administered with protein antigens

**Figure 1:**
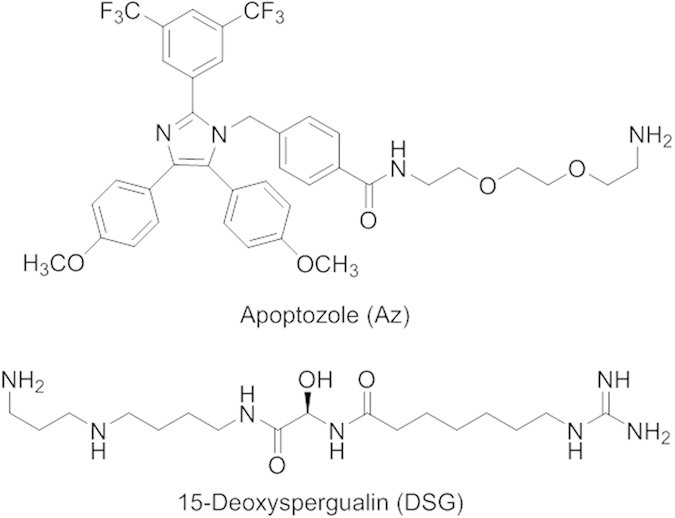
Chemical structures of Az and DSG.

In an initial study aimed at evaluating the effect of Az on the immune response to antigens, C57BL/6 mice which lack an immunoglobulin G2a (IgG2a) gene^30^ were intraperitoneally (*i.p.*) immunized with each of the two immunogenic protein antigens, keyhole limpet hemocyanin (KLH) and ovalbumin (OVA). Various amounts (0–7.55 mg/kg mouse) of Az were then *i.p.* injected daily five times from 0 to 4 days after antigen immunization. For the purpose of comparison, 15-deoxyspergualin (DSG, 12 mg/kg mouse, Fig. 1), which is known to have immunosuppressive activity^31^,^32^,^33^, was injected into mice administered with KLH under the same conditions as was Az. DSG binds to Hsc70 but not Hsp70 and it does not affect the substrate binding ability of Hsc70. Control groups were immunized with each protein antigen alone.

**Figure 2:**
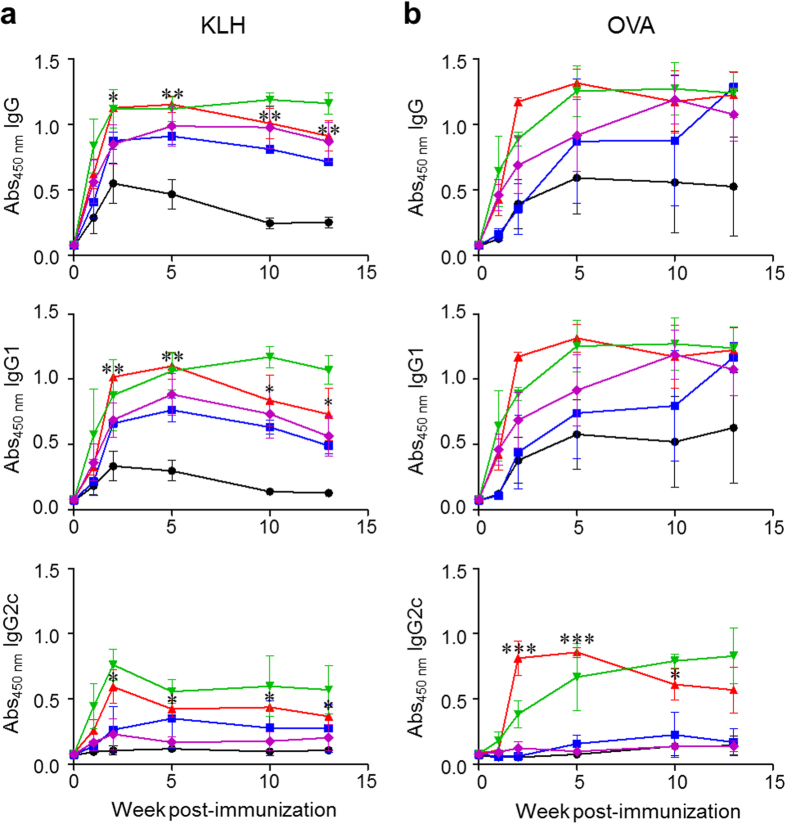
Az increases antigen-specific antibody production in a dose-dependent manner. C57BL/6 mice were *i.p.* injected with KLH (100 μg/mouse) or OVA (100 μg/mouse). The immunized mice were *i.p.* injected with various amounts of Az five times daily from 0 to 4 days after prime immunization. Sera were collected at the indicated times. Production of total IgG, IgG1 and IgG2c antibodies specific to (**a**) KLH or (**b**) OVA were determined by using an ELISA (●; Az = 0, 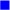; Az = 0.75, 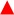; Az = 2.25, 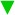; Az = 3.75, 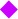; Az = 7.55 mg/kg mouse). All graphs show mean ± s.e.m. Data shown in (**a,b**) are representative of three independent experiments (*n* = 3–5 mice per group in each experiment). ^*^p < 0.05; ^**^p < 0.01; ^***^p < 0.001.

Sera were collected at different times after antigen immunization, and production of total IgG, IgG1 and IgG2c antibodies was then determined by using an ELISA. The results of immunoassays show that the injection of Az leads to an enhancement in production of total IgG and IgG1 antibodies specific to KLH or OVA compared to that of a control group untreated with Az (Fig. 2). Specifically, production of total IgG and IgG1 at 2–5 weeks after immunization increases the most when the concentrations of administered Az are 2.25–3.75 mg/kg mouse. Az treatment also leads to an increase in the production of IgG2c antibody against KLH or OVA (Fig. 2). In marked contrast to Az, DSG suppresses production of total IgG, IgG1 and IgG2c in mice administrated with a protein antigen, a phenomenon which was observed previously (Supplementary Fig. 1)^34^,^35^. Because it is known that Az inhibits Hsp70 and Hsc70 activities by binding to ATPase domains and that DSG binds to Hsc70 but not Hsp70 without affecting the substrate binding ability of Hsc70, it is reasonable that the two substances are likely to elicit conspicuously different immune responses to protein antigens.

**Figure 3:**
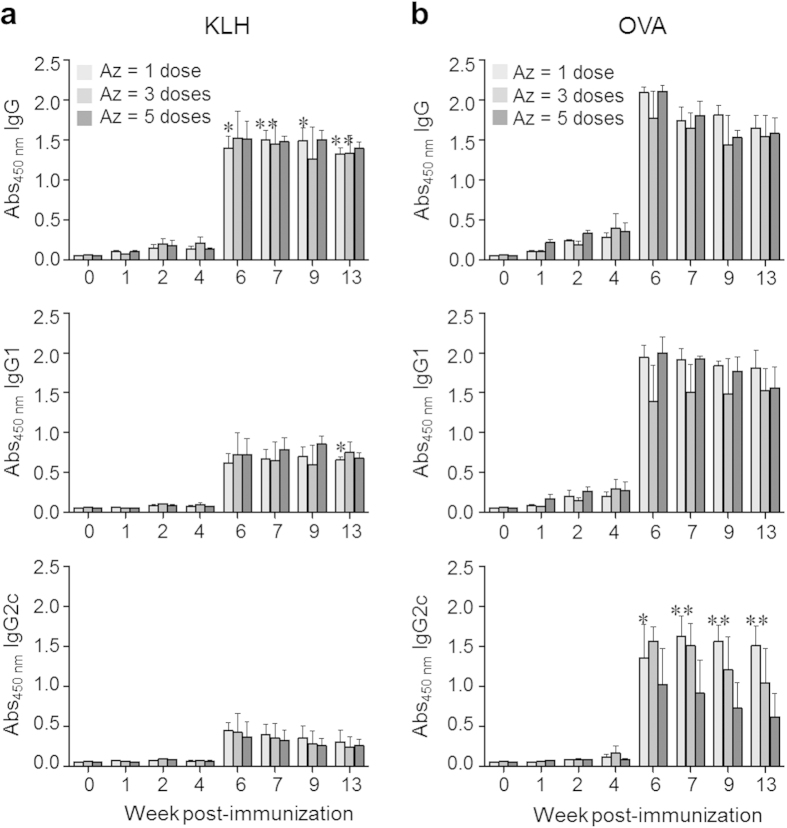
Az increases antibody production sufficiently by a single injection. C57BL/6 mice were *i.p.* injected with KLH (100 μg/mouse) or OVA (100 μg/mouse). The immunized mice were *i.p.* injected with Az (2.25 mg/kg) once (one injection after antigen immunization), three (daily injections from 0 to 2 days after antigen immunization) and five times (daily injections from 0 to 4 days after antigen immunization). Boost immunization was performed by using the same protocol as prime immunization at 4 weeks after prime immunization. Sera were collected at the indicated times. Production of total IgG, IgG1 and IgG2c antibodies specific to (**a**) KLH or (**b**) OVA was determined by using an ELISA. All graphs show mean ± s.e.m. Data shown in (**a**,**b**) are representative of three independent experiments (*n* = 3–5 mice per group in each experiment). ^*^p < 0.05; ^**^p < 0.01.

To examine the effect of the number of injections of Az on production of antibodies against administered antigens, C57BL/6 mice immunized with KLH or OVA were injected with Az (2.25 mg/kg mouse) once (one injection after antigen immunization), three (daily injections from 0 to 2 days after antigen immunization) and five times (daily injections from 0 to 4 days after antigen immunization). At 4 weeks after immunization, each group of mice was boosted by using the same protocol as in the prime immunization. Importantly, a single dose of Az was found to induce similar levels of total IgG and IgG1 production as do 3 and 5 doses (Fig. 3). The production level of an IgG2c isotype in mice immunized with KLH antigen is independent of the number of administrations of Az, but more doses of Az leads to reduced production of IgG2c specific to OVA. These findings indicate that a single administration of Az is sufficient for induction of the effective immune response.

The efficacy of Az as an adjuvant was examined by comparing its ability to enhance the immune response with those of the commercial adjuvants, Alum and PolyI:C. Alum, the most widely used inorganic adjuvant approved for clinical use in many countries, stimulates predominantly T helper type 2 (Th2) immune responses^36^. PolyI:C, a synthetic adjuvant composed of double-stranded RNA polyinosinic-polycytidylic acid, promotes mainly Th1 immune responses^37^. Th1 immune responses are involved in protection against viruses and other intracellular pathogens^38^, and Th2 responses are involved in protection against extracellular organisms and tolerance of xenografts. It is also known that IgG2 and IgG1 isotypes are generated by cytokines released by respective Th1 and Th2 cells^39^,^40^.

**Figure 4:**
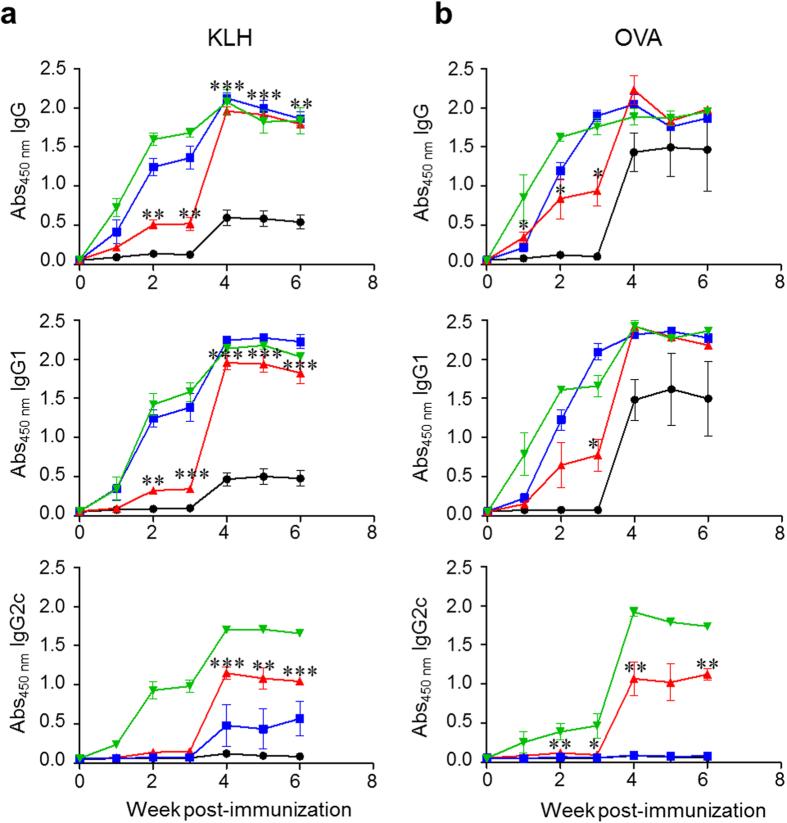
Az and known adjuvants increase antigen-specific antibody production. C57BL/6 mice were *i.p.* injected with KLH (100 μg/mouse) or OVA (100 μg/mouse). The immunized mice were *i.p.* injected once with Alum (33.0 mg/kg mouse), Az (2.25 mg/kg mouse) or PolyI:C (6.65 mg/kg mouse). Boost immunization was performed by using the same protocol as prime immunization at 3 weeks after prime immunization. Sera were collected at the indicated times. Production of total IgG, IgG1 and IgG2c antibodies specific to (**a**) KLH or (**b**) OVA was determined by using an ELISA (●; No adjuvant, 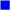; Alum, 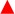; Az, 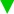; PolyI:C). Graphs show mean ± s.e.m. Data shown in (**a**,**b**) are representative of three independent experiments (*n* = 3–5 mice per group in each experiment). ^*^p < 0.05; ^**^p < 0.01; ^***^p < 0.001.

With such a consideration in mind, C57BL/6 mice immunized with KLH or OVA were injected once with Az (2.25 mg/kg mouse), Alum (33.0 mg/kg mouse) or PolyI:C (6.65 mg/kg mouse). After 3 weeks, each group of mice was boosted by using the same protocol as in the prime immunization. Sera were collected at every week after prime immunization, and the production levels of total IgG, IgG1 and IgG2c antibodies were determined by using an ELISA. The results show that immunized mice co-administered with Az, Alum or PolyI:C produce significantly increased levels of total IgG, IgG1 and IgG2c antibodies compared to control mice treated with antigen alone (Fig. 4). It was also found that the levels of total IgG and IgG1 antibodies in the Az-treated group are similarly increased to those in the Alum or PolyI:C treated groups after boost immunization. Interestingly, production of IgG2c antibody for both protein antigens is remarkably increased in the Az-treated group. The production level of IgG2c antibody in the Az-treated group was observed to be higher than that in the Alum-treated group but lower than that in the PolyI:C treated group. Taken together, the results show that Az enhances antibody production against protein antigens and thus can act as an adjuvant as do Alum and PolyI:C.

### Az activates Th1 and Th2 immune responses

**Figure 5:**
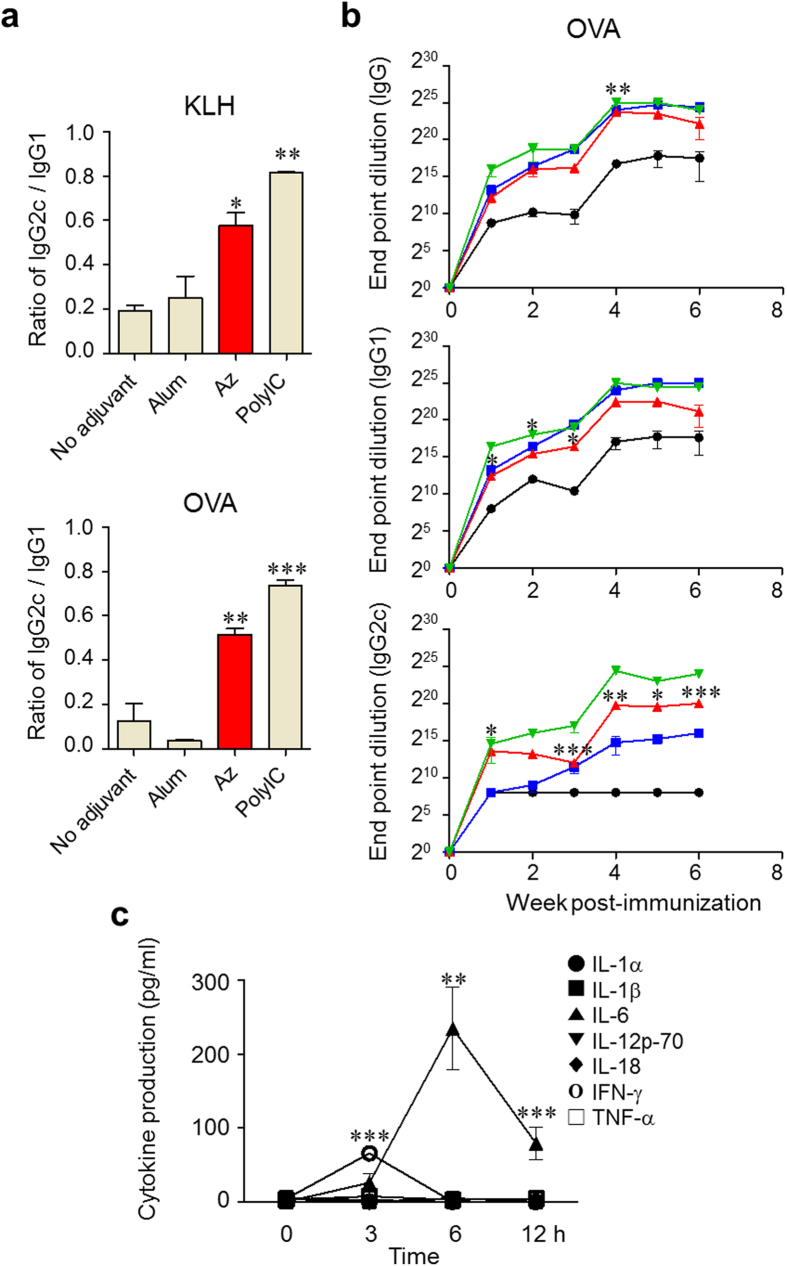
Az activates the Th1 and Th2 immune responses. (**a**) Total IgG, IgG1 and IgG2c profiles against KLH or OVA were analyzed by determining the ratio of IgG2c/IgG1 by using sera collected at 6 weeks after prime immunization (mean ± s.e.m). (**b**) For titration of antibody levels produced after OVA immunization, sera collected at 1–6 weeks after immunization were serially diluted and total IgG, IgG1 and IgG2c levels for OVA were determined (mean ± s.e.m.) (●; No adjuvant, 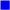; Alum, 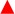; Az, 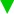; PolyI:C). (**c**) C57BL/6 mice were *i.p.* injected with Az (2.25 mg/kg) and the sera was collected at the indicated times. The amounts of cytokines were determined by using a multiplex cytokine assay. Data are representative of three independent experiments (*n* = 3–5 mice per group in each experiment). ^*^p < 0.05; ^**^p < 0.01; ^***^p < 0.001.

In order to determine whether Az activates Th1, Th2 or both immune responses, an investigation was initially conducted aimed at examining the IgG subclass distribution in immunized mice co-treated with Az. For this purpose, C57BL/6 mice immunized with KLH or OVA were injected in a single time with Az (2.25 mg/kg mouse), Alum (33.0 mg/kg) or PolyI:C (6.65 mg/kg). After 3 weeks, each group of mice was boosted by using the same protocol as in the prime immunization. Sera were collected at 6 weeks after the prime immunization, and levels of total IgG, IgG1 and IgG2c antibodies were determined by using an ELISA. The results of IgG subclass analysis show that Alum treatment leads to production of antibodies with a low IgG2c/IgG1 ratio but that PolyI:C treatment elicits generation of antibodies with a high IgG2c/IgG1 ratio, an observation that matches those previously made (Fig. 5a)^41^,^42^. The production ratio of IgG2c/IgG1 in Az-treated mice was found to be significantly higher than that in both control mice treated with antigen alone and Alum-treated group, but lower than that in the PolyI:C-treated group (Fig. 5a). The results of an end-point dilution assay of antibody against OVA also show that Az elicits a total IgG and IgG1 titer that is similar to that of Alum and PolyI:C, but an IgG2c titer that is higher than that of Alum (Fig. 5b).

We next examined the release of inflammatory cytokines in mice to further study the effect of Az on Th1 and Th2 immune responses. Sera of mice, which were administered with Az (2.25 mg/kg mouse), Alum (33.0 mg/kg) or PolyI:C (6.65 mg/kg) were collected at 0, 3, 6 and 12 h following injection. Levels of production of seven different inflammatory cytokines were determined by using a multiplex cytokine assay. Interferon-γ (IFN-γ), a critical modulator that induces Th1 cell differentiation^43^, was detected in sera collected at the early time after Az injection and interleukin-6 (IL-6), which is a main stimulator of antibody production and an inducer of Th2 differentiation^44^,^45^,^46^, was detected at a later time (Fig. 5c). When compared with the production levels of cytokines in mice treated with PolyI:C and Alum, the level of IFN-γ in Az treated mice is higher than that in Alum treated group but lower than that in PolyI:C treated group (Supplementary Fig. S2). It was also found that the level of IL-6 in Az treated mice was lower than those in mice treated with PolyI:C and Alum. We also analyzed the level of interleukin-4 (IL-4), an inducer of Th2 differentiation^47^, in sera of mice injected with Az. IL-4 was slightly increased in sera of administered mice (Supplementary Fig. S3). The results of the IgG subclass distribution and the cytokine release induced by Az suggest that this substance activates both Th1 and Th2 pathways.

### Az promotes production of cytokines in cells

It is known that inflammatory cytokines produced by antigen presenting cells (APCs), such as dendritic cells and macrophages, induce differentiation of naïve CD4 T cells into Th subset cells including Th1 and Th2 cells. In addition, the results of a previous study show that Hsp70 inhibits LPS-induced production of inflammatory cytokines by blocking NF-κB activation^27^. Based on this knowledge, we hypothesized that Az would stimulate APCs to generate inflammatory cytokines and this event would lead to enhanced production of antigen-specific antibodies in mice.

**Figure 6:**
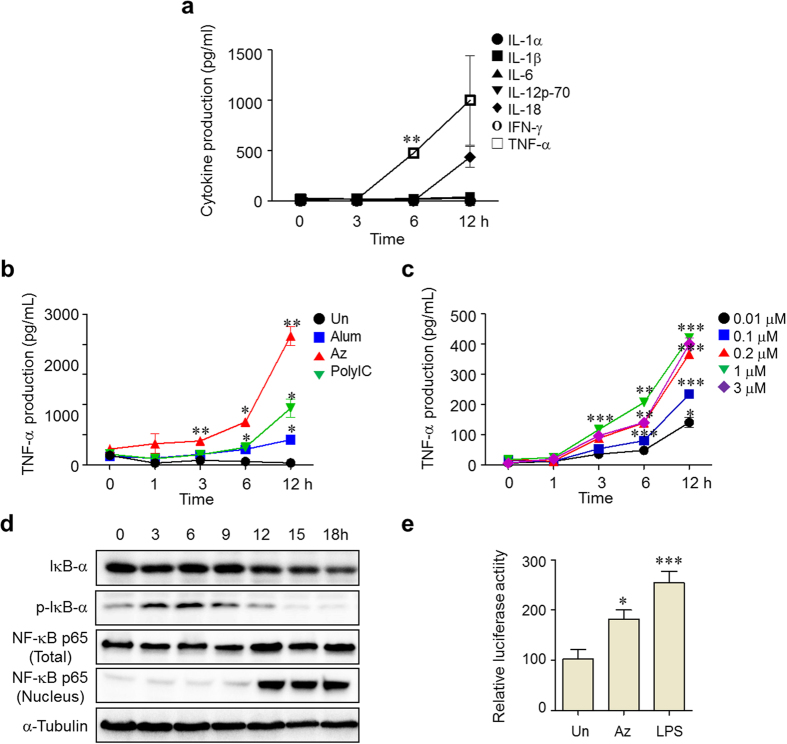
Az produces inflammatory cytokines in cells. (**a**) RAW264.7 cells were treated with 3 μM Az and the supernatant was collected at the indicated times. The amounts of cytokines were determined by using a multiplex cytokine assay. (**b**) RAW264.7 cells were treated with Az (3 μM), Alum (4 μg/ml) or PolyI:C (5 μg/ml) and supernatant was collected at the indicated times. The amount of TNF-α was determined by using an ELISA. (**c**) RAW264.7 cells were treated with various concentrations (0.01, 0.1, 0.2, 1 and 3 μM) of Az and the supernatant was collected at the indicated times. The amount of TNF-α was analyzed by using an ELISA. (**d**) RAW264.7 cells were treated with 3 μM Az and then fractionated into the cytosol and nucleus, which both were subjected to western blot analysis to detect IκBα, p-IκBα and NF-κB-p65. α-Tubulin was used as a loading control. (**e**) HUVEC cells transfected with a NF-κB luciferase reporter were treated with 3 μM Az or 1 μg/ml LPS for 12 h and then luciferase activity was measured. All graphs are shown as mean ± s.e.m. ^*^p < 0.05; ^**^p < 0.01; ^***^p < 0.001.

In order to determine if Az stimulates production of inflammatory cytokines in APCs, RAW264.7 cells, a mouse macrophage cell line, were incubated with 3 μM of Az for 3, 6 and 12 h. The released cytokines were determined by using a multiplex cytokine assay. The results reveal that Az treatment enhances the secretion of tumor necrosis factor-α (TNF-α) and IL-18 (Fig. 6a). To compare the ability of Az to produce TNF-α with those of Alum and PolyI:C, RAW264.7 cells were incubated with Az (3 μM), Alum (4 μg/ml) or PolyI:C (5 μg/ml), and then subjected to determination of TNF-α secretion. Notably, Az treatment induces more secretion of TNF-α from RAW264.7 cells than does treatment with Alum or PolyI:C (Fig. 6b). In addition, the level of TNF-α production was found to be Az dose-dependent (Fig. 6c). The results suggest that Az stimulates APCs to produce TNF-α, which subsequently mediates IFN-γ and IL-6 production from other types of immune cells in mice.

To understand the underlying mechanism by which it affects production of an inflammatory cytokine TNF-α in macrophages, the ability of Az to govern NF-κB signaling in cells was examined. This approach is based on the observation that Hsp70 blocks production of inflammatory cytokines induced by LPS through inactivation of the NF-κB pathway^27^. It is known that when the NF-κB signaling pathway is activated in cells, IκB in the cytosol is phosphorylated and it dissociates from NF-κB, which is subsequently translocated into the nucleus and binds to a specific promoter for the expression of NF-κB-dependent genes^48^,^49^,^50^,^51^. With such considerations in mind, RAW264.7 cells were exposed to Az (3 μM) for 18 h and then proteins associated with NF-κB signaling were analyzed by using western blotting. The results show that the level of a phosphorylated form of IκB in the cytosol increases until 9 h after Az treatment and then decreases gradually (Fig. 6d). On the other hand, the amount of NF-κB p65 in the nucleus increases significantly after 12 h.

To gain further information about whether translocation of NF-κB into the nucleus induces expression of a gene controlled by a NF-κB promoter, a cell line, in which luciferase expression is controlled by a NF-κB promoter, was separately incubated with Az (3 μM) or a positive control LPS (1 μg/ml) for 12 h. The results of a luciferase promoter assay show that Az activates expression of the gene controlled by a NF-κB promoter (Fig. 6e). Taken together, the results show that Az induces triggering of the NF-κB signaling pathway, thereby promoting phosphorylation of IκB in the cytosol and translocation of NF-κB into the nucleus. Activation of NF-κB signaling presumably stimulates production of inflammatory cytokines leading to an enhancement of antibody production in mice.

## Conclusions

Most small molecule inhibitors of Hsp70 and Hsc70 have been developed for use as anticancer therapeutic agents. However, the roles of these substances as modulators of the immune system have not been investigated to date. In the study described above, we uncovered evidence that Az, a small molecule inhibitor of ATPase activities of Hsp70 and Hsc70, enhances production of antibodies against immunized antigens. This finding suggests that Az can be employed as a small molecule adjuvant to enhance the efficacy of vaccinated antigens. The results of this effort also demonstrated that Az induces production of inflammatory cytokines in sera of mice and macrophage cells. Analysis of the ratio of IgG2c/IgG1 production and cytokine release patterns suggest that Az activates the Th1 and Th2 pathways. Overall, observations made in this study suggest that targeting Hsp70 and Hsc70 could represent a new strategy for discovery of small molecule-based adjuvants.

## Methods

### Synthesis of Apoptozole

Az was prepared according to the previously reported procedure^21^.

### Mice

C57BL/6 mice were purchased from the Jackson Laboratory. All mice were maintained in the specific pathogen-free facility of the YLARC at Yonsei University. All animal experiments were performed in accordance with the Korean Food and Drug Administration (KFDA) guidelines. Protocols were reviewed and approved by the Institutional Animal Care and Use Committee (IACUC) of the Yonsei Laboratory Animal Research Center (YLARC) at Yonsei University.

### Cell culture

RAW264.7 cells (mouse macrophage cell line) were plated in 96-well plate at a density of 1 × 10^6^ cells per well in culture media (DMEM supplemented with 10% fetal bovine serum (FBS), 100 units/ml penicillin and 100 μg/ml streptomycin (Gibco)). HUVECs were isolated from human umbilical cord veins by collagenase treatment, as described previously^52^,^53^, and used at passages 2 to 7. The cells were grown in M199 medium (Invitrogen) supplemented with 20% FBS, 100 units/ml penicillin, 100 μg/ml streptomycin, 3 ng/ml basic fibroblast growth factor (Upstate Biotechnology) and 5 units/ml heparin. The cells were incubated under a humidified 95%/5% (vol/vol) mixture of air at 37 °C under 5% CO_2_.

### Immunization

For immunization with antigens in all experiments, 100 μg of KLH (Sigma Aldrich) or 100 μg of OVA (Sigma Aldrich) was injected intraperitoneally (*i.p*.) into 6 week old female C57BL/6 mice. To examine the effect of Az on the immune response to protein antigens, mice immunized with KLH or OVA were *i.p.* injected with Az (0.75, 2.25, 3.75 or 7.55 mg/kg mouse) or DSG (12 mg/kg mouse) daily from 0 to 4 days after antigen immunization. Boost immunization was performed with the same protocol at 13 weeks after prime immunization.

To determine the effect of injection times of Az on the immune response, mice immunized with KLH or OVA were *i.p.* injected with Az (2.25 mg/kg) once, three times or five times daily after antigen immunization. The mice were boosted with the same protocol at 4 weeks after prime immunization. To compare the efficacy of different adjuvants on the immune response to protein antigens, mice immunized with KLH or OVA were *i.p.* injected once with of Az (2.25 mg/kg mouse), Alum (33.0 mg/kg, Pierce) or of PolyI:C (6.65 mg/kg mouse, Sigma Aldrich). At 3 weeks after prime immunization, the mice were boosted by using the same protocol as prime immunization.

### ELISA

Mice were bled for collection of sera at indicated times. The whole blood was clotted on ice for 30 min and the sera were collected from supernatant after centrifugation at 14,000 rpm at 4 °C for 15 min and stored at −80 °C for ELISA. Immunosorbant plates (96-well, Maxisorp, NUNC) were coated with 50 ng/ml KLH or 1 μg/ml OVA in 20 mM sodium bicarbonate (pH 9.6) overnight at 4 °C. The wells were washed with 0.05% PBST (0.05% Tween 20 in phosphate buffered saline, 137 mM sodium chloride, 2.7 mM potassium chloride, 10 mM sodium phosphate and 1.8 mM potassium phosphate, pH 7.4), blocked with 2.5% bovine serum albumin (BSA, Sigma Aldrich) in PBS for 2 h at room temperature, and washed 5 times with 0.05% PBST. The sera diluted with 1% BSA in PBS were added to the wells and incubated for 2 h at room temperature. The wells were washed with 0.05% PBST and then each of horseradish peroxidase (HRP)-conjugated anti-mouse IgG, IgG1 or IgG2c (Southern Biotech) diluted with 1% BSA in 0.05% PBST was added. After incubation for 1 h, the wells were washed 5 times with 0.05% PBST and then developed using Turbo TMB substrate (Pierce). The reaction was stopped by addition of 1 N sulfuric acid and the optical density at 450 nm was measured using a microplate reader (Tecan). The level of TNF-α in sera and cell culture supernatants was measured by using mouse TNF-α ELISA set (BD Bioscience) according to the manufacturer’s protocol.

### Multiplex analysis of cytokine production

Levels of IL-1α, IL-1β, IL-6, IL-12p70, IL-18, IFN-γ and TNF-α in sera and cell culture supernatants were determined by using FlowCytomix Multiple Analyte Detection System (eBioscience) according to the manufacturer’s protocol.

### Western blotting

RAW264.7 cells were treated with 3 μM Az in DMEM media for indicated time periods. Cells were washed with PBS and lysed with cytosol fractionation buffer (10 mM HEPES, pH 7.9, 10 mM KCl, 0.1 mM EDTA, 0.1 mM EGTA, 1 mM DTT and 0.5% NP-40) supplemented with a protease inhibitor cocktail (Roche). The pellets obtained by centrifugation were lysed with nucleus fractionation buffer (20 mM HEPES, pH 7.9, 400 mM NaCl, 1 mM EDTA, 1 mM EGTA and 1 mM DTT) supplemented with a protease inhibitor cocktail. After incubation on ice for 30 min, the supernatant was collected by centrifugation. Protein samples were loaded and run on 13% SDS-PAGE gel. The proteins were transferred to PVDF membrane (Pall), which was then blocked with 5% skim milk in 0.1% TBST (0.1% Tween20, 150 mM sodium chloride, 50 mM Tris-HCl, pH 7.4). The membrane was blotted by using antibodies (anti-IκBα, anti-p-IκBα, anti-NFκB, anti-α-tubulin and anti-LaminA/C (Santacruz)) at 1:2000 diluted in 0.1% TBST and secondary antibodies (HRP-conjugated anti-mouse, anti-rabbit antibodies (Santacruz)). To determine the band size, a pre-stained size marker (Intron biotechnology) was used. The specific bands were detected using Westzol ECL solution.

### Dual luciferase reporter assay

HUVECs were transfected with NF-κB luciferase reporter constructs and pRL-CMV for normalization using Lipofectamine according to the manufacturer’s instructions (Invitrogen). After 24 h, HUVECs were lysed with passive lysis buffer and luciferase activity was measured by using the Dual-Luciferase Reporter Assay System (Promega).

### Data analysis

Statistical analysis was performed with two-tailed unpaired Student t tests by using Prism software version 5.02 (GraphPad).

## Additional Information

**How to cite this article**: Baek, K.-H. *et al.* A small molecule inhibitor for ATPase activity of Hsp70 and Hsc70 enhances the immune response to protein antigens. *Sci. Rep.* **5**, 17642; doi: 10.1038/srep17642 (2015).

## Supplementary Material

Supplementary Information
