# Supplementary material for: A small molecule inhibitor for ATPase activity of Hsp70 and Hsc70 enhances the immune response to protein antigens
Source: Sci Rep. 2015 Dec 3;5:17642. doi: 10.1038/srep17642 (PMC4668564; doi:10.1038/srep17642)
Supplement: Supplementary Information [file srep17642-s1.pdf]

SUPPORTING INFORMATION

A small molecule inhibitor for ATPase activity of Hsp70 and Hsc70 enhances the immune response to protein antigens

Kyung-Hwa Baek,[1] Haiying Zhang,[2] Bo Ryeong Lee,[2] Young-Guen Kwon,[2] Sang-Jun Ha,[2,*] Injae Shin[1,*]

[1]National Creative Research Initiative Center for Biofunctional Molecules, Department of Chemistry, Yonsei University, Seoul 03722, Korea

[2]Department of Biochemistry, College of Life Science & Biotechnology, Yonsei University, Seoul 03722, Korea

*Corresponding authors: injae@yonsei.ac.kr (I. Shin) or sjha@yonsei.ac.kr (S.-J. Ha)

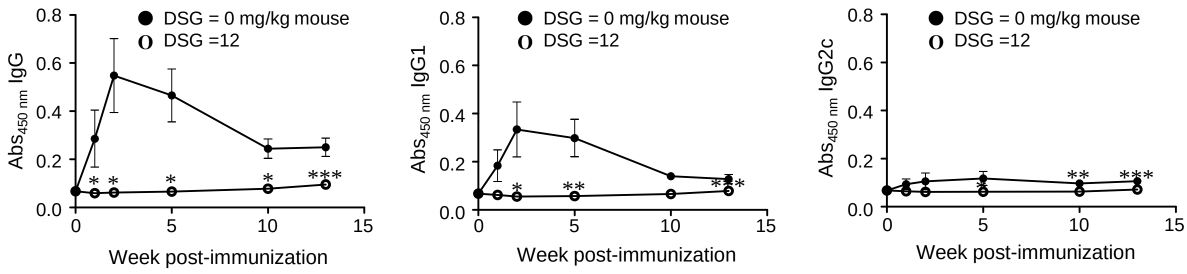

Figure S1. DSG suppresses the immune response to a protein antigen. C57BL/6 mice (n = 3-5 per group) were *i.p.* injected with KLH (100 μg/mouse). The immunized mice were *i.p.* injected with DSG (12 mg/kg mouse) five times daily from 0 to 4 days after prime immunization. Sera were collected at the indicated times. Production of total IgG, IgG1 and IgG2c antibodies specific to KLH was determined by using an ELISA (mean ± s.e.m, n = 3-5 mice per group). *, $p < 0.05$; **, $p < 0.01$; ***, $p < 0.001$.

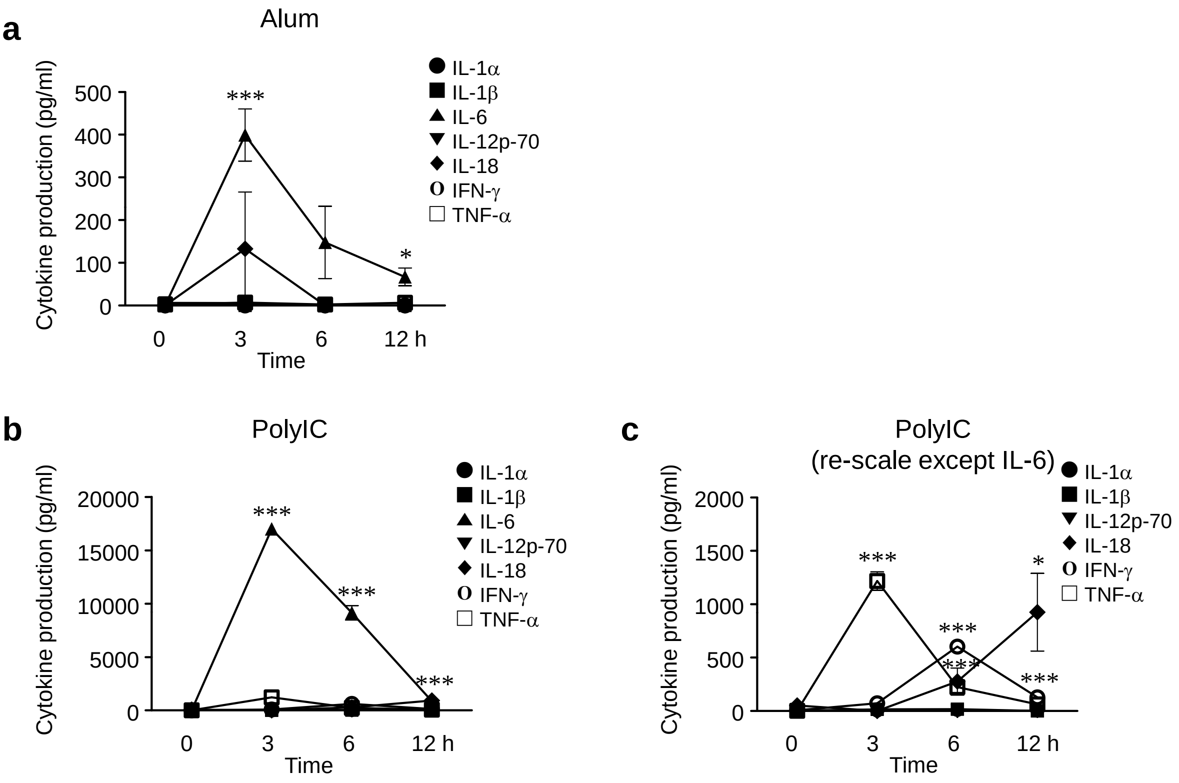

Figure S2. Alum and PolyI:C produce inflammatory cytokines in mice. C57BL/6 mice (*n* = 3-5 per group) were *i.p.* injected with (a) Alum (33.0 mg/kg mouse) and (b) PolyI:C (6.65 mg/kg mouse), and sera were collected at the indicated times. The amounts of cytokines were determined by using a multiple cytokine assay. (c) Re-scaled graph of graph (b). All graphs are shown as mean ± s.e.m. *, $p < 0.05$; **, $p < 0.01$; ***, $p < 0.001$.

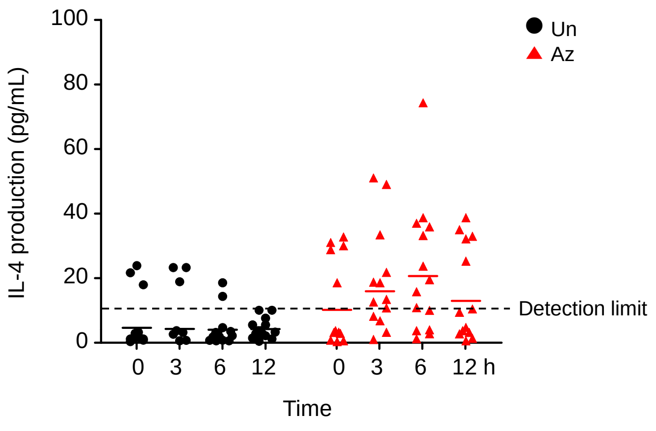

Figure S3. Az slightly increases IL-4 production in mice. C57BL/6 mice (n = 15 per group) were *i.p.* injected with Az (2.25 mg/kg mouse) and sera were collected at the indicated times. The amount of IL-4 was determined by using an ELISA assay (mean ± s.e.m).
